# ﻿From surface to caves: new species of *Diploexochus* Brandt, 1833 (Oniscidea, Armadillidae) from Colombia, with the description of the first troglobitic species

**DOI:** 10.3897/zookeys.1223.133267

**Published:** 2025-01-13

**Authors:** Ricardo Borja-Arrieta, Carlos Mario López-Orozco, Yesenia M. Carpio-Díaz, Miguel Gutierrez-Estrada, Ivanklin Soares Campos-Filho, Gabriel R. Navas-S., Maria Elina Bichuette

**Affiliations:** 1 Laboratório de Estudos Subterrâneos, Universidade Federal de São Carlos, São Carlos, São Paulo, Brazil; 2 Grupo de Investigación en Artrópodos Neotropicales, Universidad de Cartagena, Programa de Biología, Campus San Pablo, Cartagena de Indias, Colombia; 3 Grupo de Investigación Biología Descriptiva y Aplicada, Universidad de Cartagena, Programa de Biología, Campus San Pablo, Cartagena de Indias, Colombia; 4 Grupo de Investigación Hidrobiología, Universidad de Cartagena, Programa de Biología, Campus San Pablo, Cartagena de Indias, Colombia; 5 Grupo de Investigación Territorios Semiáridos del Caribe, Facultad de Ingeniería, Universidad de la Guajira, La Guajira, Colombia; 6 Department of Biological Sciences, University of Cyprus, Lefkosia (Nicosia), Cyprus

**Keywords:** Cave-dwelling, Colombian Caribbean, Neotropical, subterranean systems, terrestrial isopods, Tropical Dry Forest

## Abstract

Two new species of *Diploexochus* are described, *Diploexochuscacique***sp. nov.** from Cerro Bañaderos in Hatonuevo, La Guajira, and *Diploexochustroglobius***sp. nov.** from Roca Madre Cave, Toluviejo, Sucre, both from the Tropical Dry Forest (TDF) areas of the Colombian Caribbean. The latter represent the first troglobitic species of the genus. Moreover, based on specimens from Sierra Nevada de Santa Marta, Magdalena (type locality), *Venezillobrevispinis* is placed into *Diploexochus* and an identification key for all species of the genus is given. The present work describes the first troglobitic species of the genus, and expand the knowledge of its distribution in northern South America.

## ﻿Introduction

Terrestrial isopods (Oniscidea) are considered one of the most diverse groups of crustaceans, comprising approximately 4,000 species in more than 500 genera in 38 or 39 families, distributed in almost all terrestrial habitats, including caves (Schmalfuss 2003; Hornung 2011; Sfenthourakis and Taiti 2015; Campos-Filho et al. 2017a, 2018, 2023a, 2023b; Taiti 2017; Dimitriou et al. 2019; Campos-Filho and Taiti 2021). Within Oniscidea, the family Armadillidae Brandt, 1831 is the most diverse, including more than 600 species in 81 genera and distributed in Neotropical, Afrotropical, Oriental, and Australian regions (Taiti et al. 1998; Schmalfuss 2003; Sfenthourakis and Taiti 2015; Rodríguez-Cabrera and Armas 2023). In Colombia, 13 species of the family are known: *Ctenorillobinomio* Carpio-Díaz, Bichuette & Campos-Filho, 2023, *C.dazai* Carpio-Díaz, López-Orozco & Campos-Filho, 2018, *C.humboldti* Carpio-Díaz, López-Orozco & Campos-Filho, 2023, *C.mincaensis* López-Orozco, Carpio-Díaz & Campos-Filho, 2023, *C.orientalis* Carpio-Díaz, Taiti & López-Orozco, 2023, *C.papagayoensis* Carpio-Díaz, Borja-Arrieta & Campos-Filho, 2023, *C.tayrona* López-Orozco, Borja-Arrieta & Campos-Filho, 2023, *C.tuberosus* (Budde-Lund, 1904), *Synarmadilloruthveni* (Pearse, 1915), *Venezillobrevispinis* (Pearse, 1915), *V.gigas* (Miers, 1877), *V.grenadensis* (Budde-Lund, 1893), and *V.vincentis* (Budde-Lund, 1904) (Richardson 1912; Pearse 1915; Carpio-Díaz et al. 2018, 2023a; López-Orozco et al. 2022).

The genus *Diploexochus* Brandt, 1833 comprises five species exclusively distributed in South America, i.e., *D.echinatus* Brandt, 1833 from Brazil, French Guiana, Guyana, and Trinidad, *D.obscurus* Cardoso, Bastos-Pereira & Ferreira, 2022, *D.spinatus* Cardoso, Bastos-Pereira & Ferreira, 2022, *D.carrapicho* Campos-Filho, López-Orozco & Taiti, 2023, and *D.exu* Campos-Filho, Sfenthourakis & Bichuette, 2023 from Brazil (Schmalfuss 2003; Campos-Filho et al. 2023a; Cardoso et al. 2023). The genus is characterized by the shape and direction of the pereonites 1–7 epimera and pleonites 3–5 epimera, large schisma of the pereonite 1 epimera, frontal shield surpassing the vertex of the cephalon, and the presence of well-developed dorsal tubercles (see Campos-Filho et al. 2017a).

According to the Sector Technical Standard NTS-AV012 of 2008, a cavern is defined as any subterranean space within rocks large enough for human entry; it may have been formed in rocks or ice and may be filled with water, sediments, blocks, lava, and sometimes it may be impenetrable. The environmental conditions in these habitats are stable and support the establishment of various forms of life, including troglobitic organisms. These organisms are characterized by completing their entire life cycle within caves and exhibit high degree of troglomorphism (Galán and Herrera 1998). Various subterranean systems have been reported in Colombia, especially for the department of Santander, where most of the studies are concentrated (Muñoz-Saba et al. 1998a, 1998b; 2013; Castellanos-Morales et al. 2015; Barriga et al. 2019; Valdivieso 2022). Despite this, the associated diversity for most caves is still only estimated, particularly in the Caribbean region, where the number of subterranean systems remains unknown.

The diversity of Oniscidea from Colombia has increased considerably in the last years (López-Orozco et al. 2014, 2016, 2017, 2022; Carpio-Díaz et al. 2016, 2018, 2021, 2023a, 2023b; Campos-Filho et al. 2020). However, this knowledge is far from complete, considering the territorial extension of the country which difficult extensive surveys. In this study, two new species of *Diploexochus* are described for the department of La Guajira and Sucre. Moreover, the examination of specimens of *V.brevispinis* from Sierra Nevada de Santa Marta, Magdalena Department (type locality), allowed the placement of the species into the genus *Diploexochus*. Additionally, an identification key for the species and ecological and conservation remarks are provided.

## ﻿Materials and methods

The specimens were preserved in 70% ethanol. Identifications were based on morphological characters using micropreparations in Hoyer’s medium (Anderson 1954). Illustrations were made with aid of a camera lucida mounted on Wild M3 and M20 microscopes. The images of the species were obtained using a stereomicroscope SteREO Discovery.V12 ZEISS with an adapted camera Axiocam ERc 5s. The final illustrations were created using GIMP software (v. 2.8) following the method proposed by Montesanto (2015, 2016). Respiratory structures were classified according to Paoli et al. (2002). The examined material is deposited in the Collection of the University of Cartagena, Cartagena, Colombia (CBUDC-CRU).

## ﻿Results

### ﻿Systematics

**Suborder Oniscidea Latreille, 1802**

**Family Armadillidae Brandt, 1831**

#### ﻿Genus Diploexochus

Taxon classificationAnimaliaIsopodaArmadillidae

Brandt, 1833

DDD89723-2694-5EB1-835C-7E09E364EC59

##### Type species.

*Diploexochusechinatus* Brandt, 1833, by monotypy (see Schmidt and Leistikow 2004).

#### ﻿ Diploexochus brevispinis

Taxon classificationAnimaliaIsopodaArmadillidae

(Pearse, 1915) comb. nov.

D66510F7-C249-56A2-9113-10FF7BACEAD8

**Figure 1.**
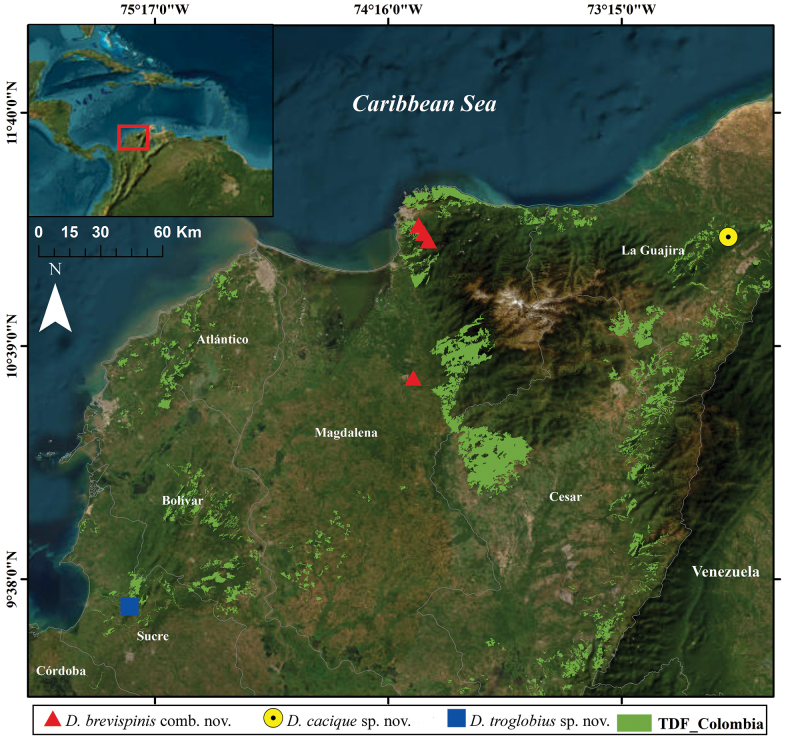
Distribution map of the *Diploexochus* species from Colombia. Green areas: Tropical Dry Forest (TDF) from Colombia (García et al. 2014).

**Figure 2.**
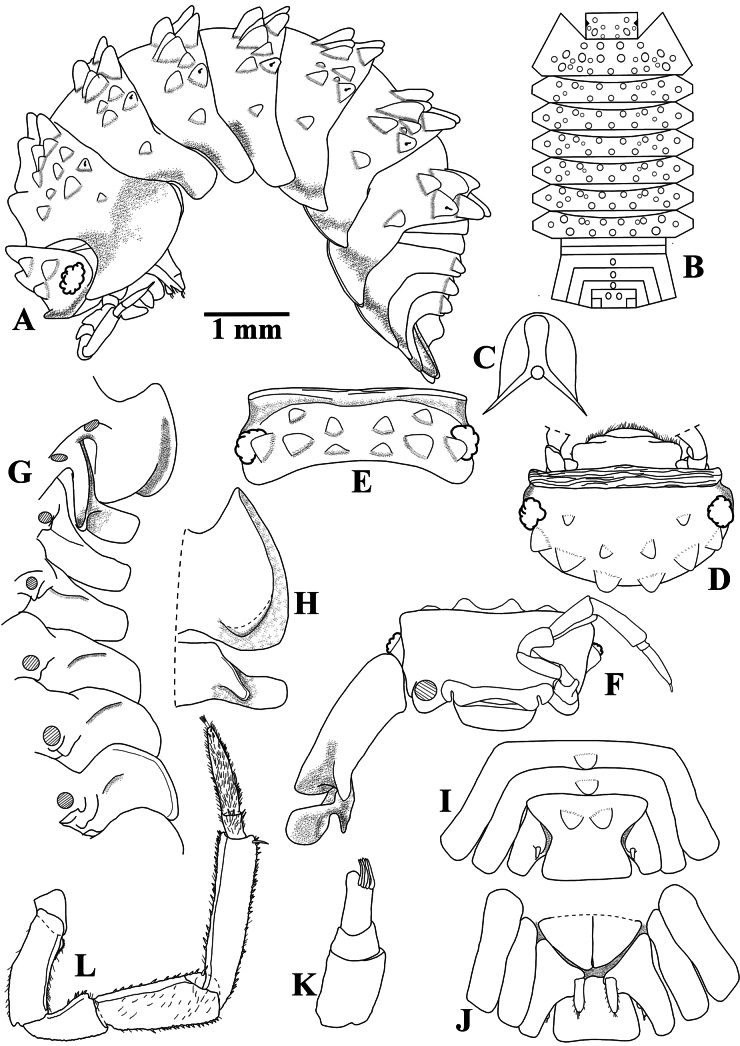
*Diploexochusbrevispinis* (Pearse, 1915), comb. nov. (♀ CBUDC-CRU 344) **A** habitus, lateral view **B** dorsal tubercles scheme **C** dorsal scale-seta **D** cephalon, dorsal view **E** cephalon, posterior view **F** cephalon and pereonites 1 and 2, frontal view **G** pereonites 1–7 epimera, ventral view **H** pereonites 1 and 2 epimera, ventral view **I** pleonites 4 and 5, telson, and uropods, dorsal view **J** pleonites 4 and 5, telson, and uropods, ventral view **K** antennula **L** antenna.

**Figure 3.**
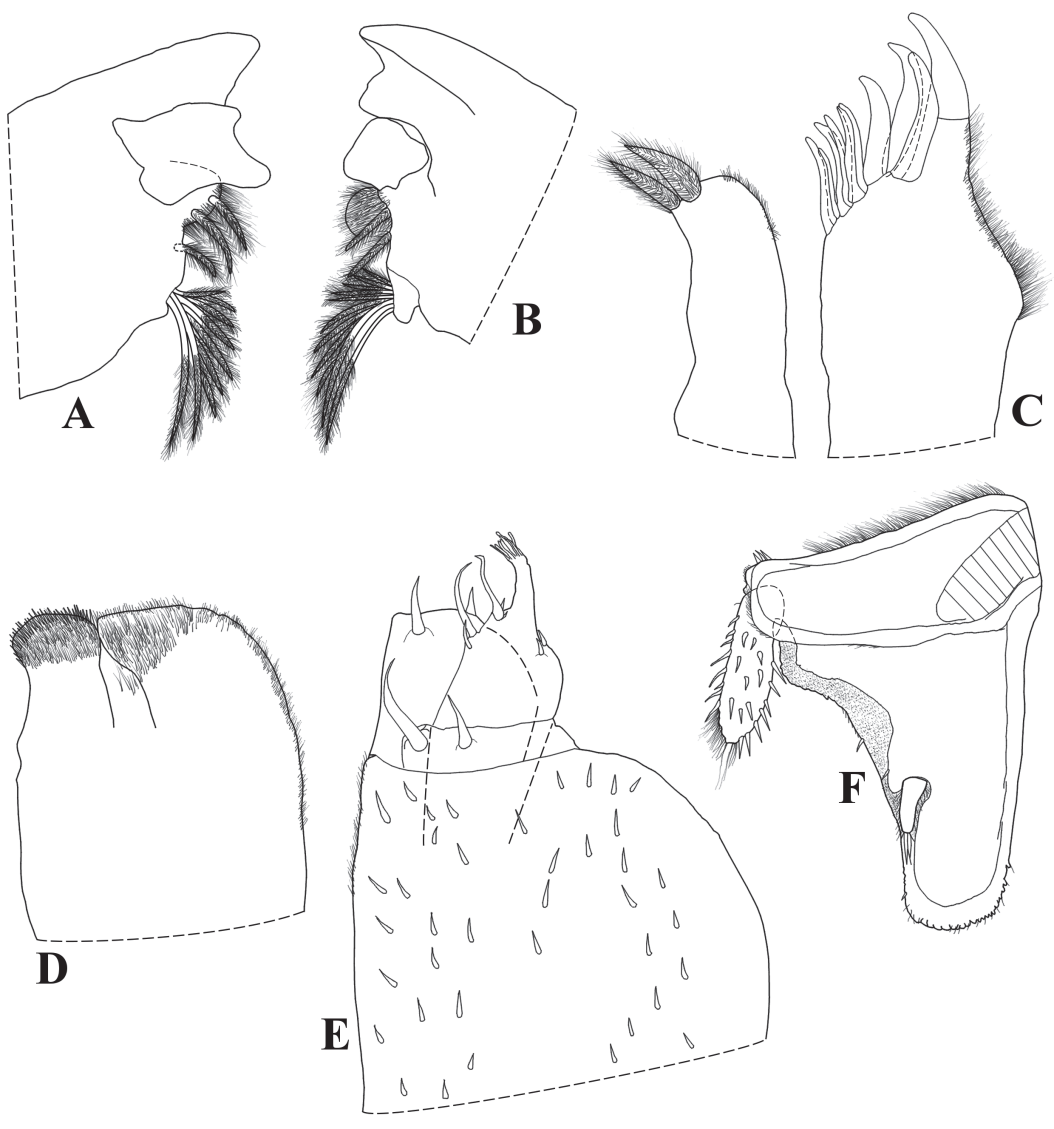
*Diploexochusbrevispinis* (Pearse, 1915), comb. nov. (♀ CBUDC-CRU 344) **A** left mandible **B** right mandible **C** maxillula **D** maxilla **E** maxilliped **F** uropod.

**Figure 4.**
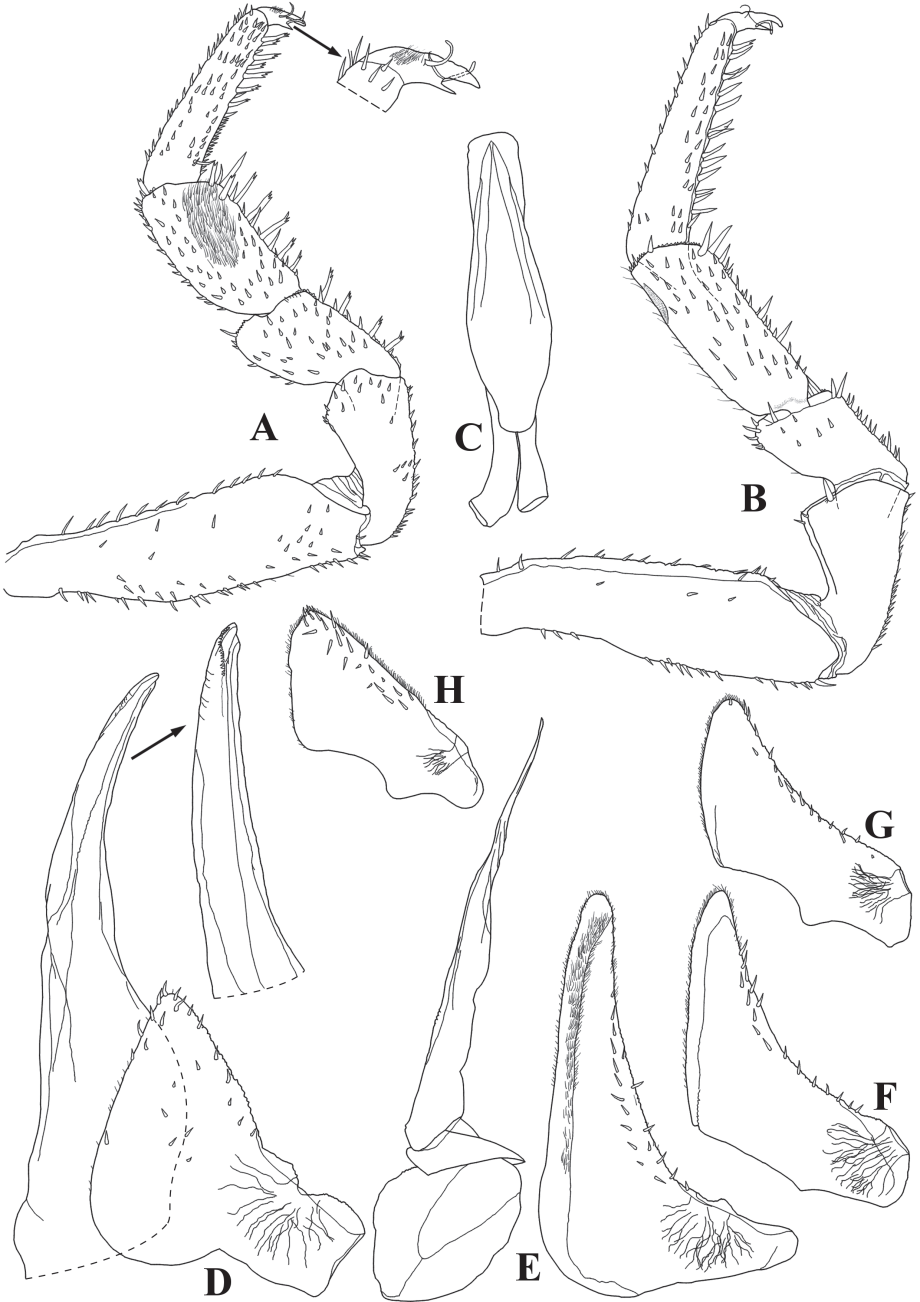
*Diploexochusbrevispinis* (Pearse, 1915), comb. nov. male (♂ CBUDC-CRU 344) **A** pereopod 1 **B** pereopod 7 **C** genital papilla **D** pleopod 1 **E** pleopod 2 **F** pleopod 3 exopod **G** pleopod 4 exopod **H** pleopod 5 exopod.

Figs 1 , 2 , 3 , 4 , 8A , 13A

Cubaris brevispinis Pearse, 1915: 543, fig. 5.
Cubaris brevispinis : Van Name 1936: 382, fig. 232.
Venezillo (Vandelillo) brevispinis : Arcangeli 1957: 121.
Venezillo brevispinis : Leistikow and Wägele 1999: 47; Schmalfuss 2003: 286.

##### Material examined.

Colombia • 1♂, 1♀ (parts in micropreparations), Hacienda Cafetera Cincinati, Sierra Nevada de Santa Marta, Santa Marta, Magdalena, 11°6'34.14"N, 74°5'30.84"W, leg. CM López-Orozco, YM Carpio-Díaz, 13.VIII. 2018, CBUDC-CRU 344 • 3♂, 4♀, same locality and collectors as for preceding, CBUDC-CRU 343.

##### Redescription.

Maximum body length: male 7 mm, female 7.5 mm. Color dark brown, cephalon, pereon, pleon, and telson strongly pigmented, pleonites 3–5 epimera less pigmented (Fig. 8A); upper portion of tubercles, pereonite 1 epimera anterior and posterior corners, pereonites 2, 3, and 6 epimera weakly pigmented, sometimes depigmented. Color pattern preserved in ethanol (Figs 2A, 13A). Body in lateral view as in Fig. 2A. Endoantennal conglobation (Figs 2A, 13A). Dorsum covered with large triangular tubercles, arranged as follows (Fig. 2A, B): vertex of cephalon with 10 tubercles in three rows; pereonite 1 with 25–29 tubercles; pereonites 2–6 with 17 tubercles; pereonite 7 with 15 tubercles; pleonites 3–5 with one tubercle on median portion, and telson with two paramedian tubercles. Pereonites 1–7 epimera with one line of ***noduli laterales*** per side inserted on outer surface of second tubercle of posterior row (Fig. 2A). Dorsal surface with short semi-circular scale-setae (Fig. 2C). Cephalon (Fig. 2D–F) with frontal shield prominent, distinctly protruding above vertex; eyes of 16 ommatidia. Pereonites 1–7 epimera flattened and directed outwards; pereonite 1 strongly grooved on lateral margin, inner lobe of schisma rounded, not extending beyond posterior margin of outer lobe (Fig. 2G–H), pereonite 2 with triangular ventral lobe obliquely directed backwards; pereonites 3–7 with oblique ventral ridge (Fig. 2A–G). Pleonites 3–5 (Fig. 2I, J) with epimera well developed, rectangular, and directed outwards. Telson (Fig. 2I) with proximal part slightly broader than distal part, dorsum slightly depressed, distal margin straight. Antennula (Fig. 2K) of three articles, proximal article longest, distal article with five aesthetascs inserted apically. Antenna (Fig. 2L) short, not surpassing posterior margin of pereonite 1 when extended backwards; flagellum of two articles, distal article about three times as long as first bearing one row of two lateral aesthetascs. Mandibles with molar penicil dichotomized; left mandible (Fig. 3A) with 2+1 penicils, right mandible (Fig. 3B) with 1+1 penicils. Maxillula (Fig. 3C) inner endite with two stout penicils; outer endite with 4+6 simple teeth. Maxilla (Fig. 3D) inner lobe rounded and covered with thick setae; outer lobe rounded, twice as wide as inner lobe, covered with thin setae. Maxilliped (Fig. 3E) basis rectangular bearing sparse setae; palp with two distinct setae on basal article; endite subrectangular, medial seta overpassing distal margin, distal margin with one short seta. Pereopods 1–7 merus and carpus with sparse setae on sternal margin; carpus 1 with distal setae cleft at apex; ungual seta and dactylar organ simple. Uropod (Fig. 3F) protopod flattened, enlarged on basal part, distal part subrectangular, medial margin slightly concave; exopod short, inserted dorsally near medial margin below distinct lobe, lobe not extending beyond medial margin. Pleopod exopods 1–5 with monospiracular respiratory structures.

**Figure 5.**
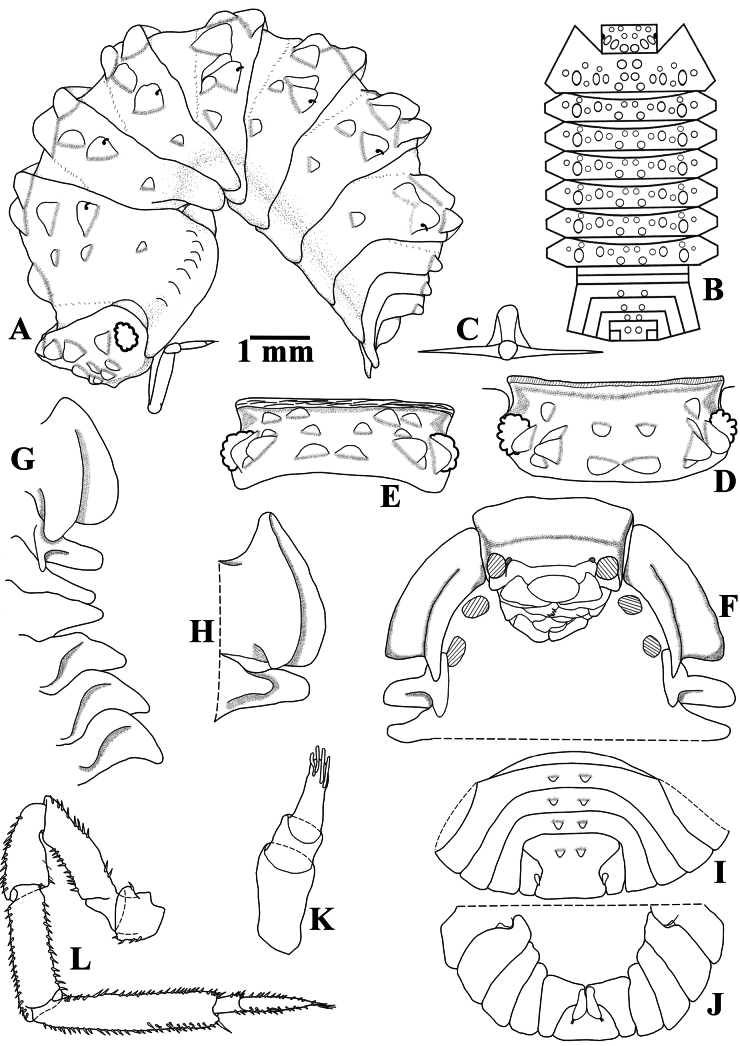
*Diploexochuscacique* López-Orozco, Carpio-Díaz & Campos-Filho, sp. nov. (♀ paratype, CBUDC-CRU 413) **A** habitus, lateral view **B** dorsal tubercles scheme **C** dorsal scale-seta **D** cephalon, dorsal view **E** cephalon, posterior view **F** cephalon and pereonites 1–3, frontal view **G** pereonites 1–7 epimera, ventral view **H** pereonites 1 and 2 epimera, ventral view **I** peonites 3–5, telson, and uropods, dorsal view **J** pleonites 3–5, telson, and uropods, ventral view **K** antennula **L** antenna.

**Male.** Pereopods 1–7 (Fig. 4A, B) without particular modifications. Genital papilla as in Fig. 5C. Pleopod 1 (Fig. 4D) exopod triangular, wider than long, outer and inner margin bearing many small setae, distal part triangular, proximal outer part quadrangular; endopod about twice as long as exopod, distal portion slightly directed outwards. Pleopod 2 (Fig. 4E) exopod triangular, outer margin strongly concave bearing many setae; endopod longer than exopod. Pleopod 3–5 exopods as in Fig. 4F–H.

##### Remarks.

Pearse (1915) described *Cubarisbrevispinis* from Minca, Sierra Nevada de Santa Marta, Colombia. Vandel (1952) proposed new morphological characters for the genus *Venezillo*, such as the shape of the epimera and ventral lobes of pereonites 1 and 2. Arcangeli (1957), based on the previous characters, transferred *C.brevispinis* to the genus *Venezillo*, at that time within the subgenus Vandelillo.

Among the characteristics mentioned by Pearse (1915), the number and arrangement of the dorsal tubercles of the cephalon, pereonites 2–7, pleon, and telson, the shape and direction of the pereonites 1–7 epimera, and the number of ommatidia were confirmed here. The only distinct characteristic contrasting with the original description was the number of tubercles on pereonite 1. Pearse reported 29 tubercles, while 25 were observed in the present study. Probably, this difference is related with the size of the specimens. Additionally, in the drawings of Pearse, the lateral schisma of the pereonite 1 epimera reached about half of its length, which was also confirmed here [see Fig. 2F–H for comparison with Pearse (1915)]. Thus, based on the generic diagnostic characters mentioned previously, *V.brevispinis* is placed into the genus *Diploexochus*.

*Diploexochusbrevispinis* comb. nov. easily differs from *D.carrapicho*, *D.echinatus*, *D.exu*, *D.obscurus*, and *D.spinatus* in the number and arrangement of the dorsal tubercles of the cephalon, pereon, and pleon. Moreover, it differs in having the antennula with five distal aesthetascs (vs six in *D.exu* and *D.carrapicho*, 10 in *D.echinatus*, seven in *D.obscurus*, and nine in *D.spinatus*), mandibles with dichotomized molar penicil (vs simple in all species), and uropod protopod with median lobe not protruding beyond the medial margin (vs protruding in all species) (see Campos-Filho et al. 2017a, 2023a; Cardoso et al. 2023).

##### Natural history.

Specimens of *Diploexochusbrevispinis* comb. nov. were collected under fallen logs in a sub-Andean forest close to the road at the Cincinnati farm in the Sierra Nevada de Santa Marta, Magdalena, Colombia (Fig. 8A).

##### Distribution.

This species is known only from its type locality in Tropical Dry Forest (TDF) and Andean forest of Sierra Nevada de Santa Marta (Fig. 1).

#### ﻿ Diploexochus cacique

Taxon classificationAnimaliaIsopodaArmadillidae

López-Orozco, Carpio-Díaz & Campos-Filho sp. nov.

A3B0B48A-9AFE-52E9-A07A-D8B88DC400DB

https://zoobank.org/3BD3757B-7DFF-4554-B456-09AFC7AF3E2E

**Figure 6.**
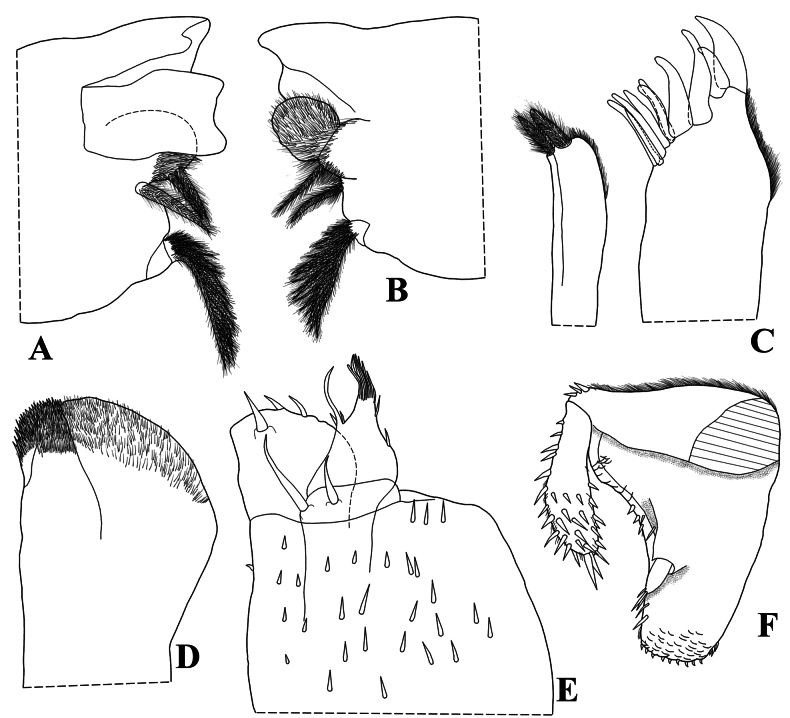
*Diploexochuscacique* López-Orozco, Carpio-Díaz & Campos-Filho, sp. nov. (♀ paratype, CBUDC-CRU 413) **A** right mandible **B** left mandible **C** maxillula **D** maxilla **E** maxilliped **F** uropod.

**Figure 7.**
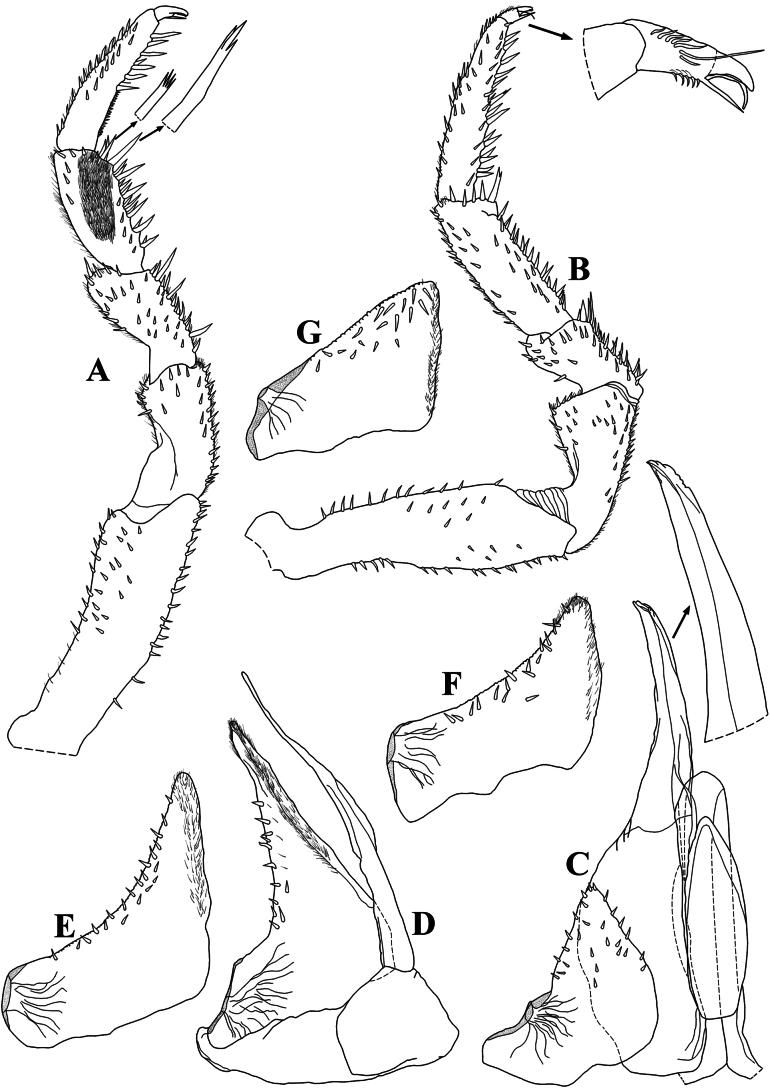
*Diploexochuscacique* López-Orozco, Carpio-Díaz & Campos-Filho, sp. nov. (♂ paratype, CBUDC-CRU 413) **A** pereopod 1 **B** pereopod 7 **C** pleopod 1 and genital papilla **D** pleopod 2 **E** pleopod 3 exopod **F** pleopod 4 exopod **G** pleopod 5 exopod.

**Figure 8.**
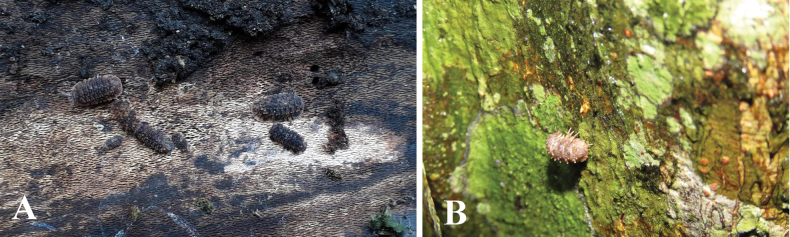
**A***Diploexochusbrevispinis* (Pearse, 1915), comb. nov. under fallen tree in the Sierra Nevada de Santa Marta, Magdalena **B***Diploexochuscacique* López-Orozco, Carpio-Díaz & Campos-Filho, sp. nov. on the tree bark, Cerro Bañaderos, Hatonuevo, La Guajira.

Figs 1 , 5 , 6 , 7 , 8B , 13B

##### Type material.

Colombia • 1♂, ***holotype***, Cerro Bañaderos, Hatonuevo, La Guajira, 11°7'33.3"N, 72°47'6.9"W, 12.I.2016, leg. M Gutierrez-Estrada, CBUC-CRU 350 • 1♂,1♀ (parts in micropreparations), ***paratypes***, same data as holotype, CBUDC-CRU 413 • 1♀, ***paratypes***, same data as holotype, CBUDC-CRU 414.

##### Description.

Maximum body length: male 8 mm, female 9 mm. Body outline as in Fig. 5A. Color dark brown with typical muscular insertions (Figs 8B, 13B); upper portion of tubercles randomly depigmented; pereonite 1 epimera anterior corner, pereonites 2–7 paramedian portions, pereonites 3 and 4 epimera, and pleonites 3–5 epimera randomly depigmented. Endoantennal conglobation (Figs 5A, 8B, 13B). Dorsum covered with large triangular tubercles, arranged as follows (Fig. 5A, B): vertex of cephalon with 12 tubercles in three rows; pereonite 1 with 20 tubercles; pereonites 2–6 with 16 tubercles; pereonite 7 with 15 tubercles; pleonites 3–5 and telson with two paramedian tubercles. Pereonites 1–7 epimera with one line of ***noduli laterales*** per side inserted on outer surface of second tubercle of posterior row (Fig. 5A). Dorsal surface with short and narrow semi-circular scale-setae (Fig. 5C). Cephalon (Fig. 5D–F) with frontal shield prominent, distinctly protruding above vertex; eyes with 20–21 ommatidia. Pereonites 1–7 epimera flattened and slightly directed outwards; pereonite 1 strongly grooved on lateral margin, inner lobe of schisma rounded, extending beyond posterior margin of outer lobe (Fig. 5G, H), pereonite 2 with triangular and narrow ventral lobe directed outwards, not extending beyond posterior margin of epimera; pereonites 4–7 with oblique ventral ridge (Fig. 5F–H). Pleonites 3–5 (Fig. 5I, J) with epimera well developed, rectangular and slightly directed outwards. Telson (Fig. 5I) with proximal part slightly broader than distal part, dorsum slightly depressed, distal margin straight. Antennula (Fig. 5K) of three articles, proximal and distal articles subequal in length, distal article with four aesthetascs inserted sub-apically. Antenna (Fig. 5L) short, not surpassing posterior margin of pereonite 1 when extended backwards; flagellum of two articles, distal article about twice as long as first bearing one row of two lateral aesthetascs. Mandibles with molar penicil semi-dichotomized; left mandible (Fig. 6A) with 2+1 penicils, right mandible (Fig. 6B) with 1+1 penicils. Maxillula (Fig. 6C) inner endite with two stout penicils, distal margin bearing fringe of thin setae; outer endite of 4+6 teeth simple. Maxilla (Fig. 6D) inner lobe rounded covered with thick setae; outer lobe rounded, three times as wide as inner lobe, covered with thin setae. Maxilliped (Fig. 6E) basis rectangular bearing sparse setae; palp with two distinct setae on basal article; endite subrectangular, medial seta overpassing distal margin, distal margin bearing two setae. Pereopods 1–7 merus and carpus bearing setae on sternal margin, not sparse appearance; carpus 1 with distal setae cleft at apex; ungual seta and dactylar organ simple, both surpassing outer claw. Uropod (Fig. 6F) protopod flattened, enlarged on basal part, distal part subrectangular, medial margin concave; exopod short inserted dorsally near medial margin bellow distinct lobe, lobe not extending beyond medial margin; endopod club-shaped bearing many short setae on distal part. Pleopods 1–5 exopods with monospiracular respiratory structures.

**Figure 9.**
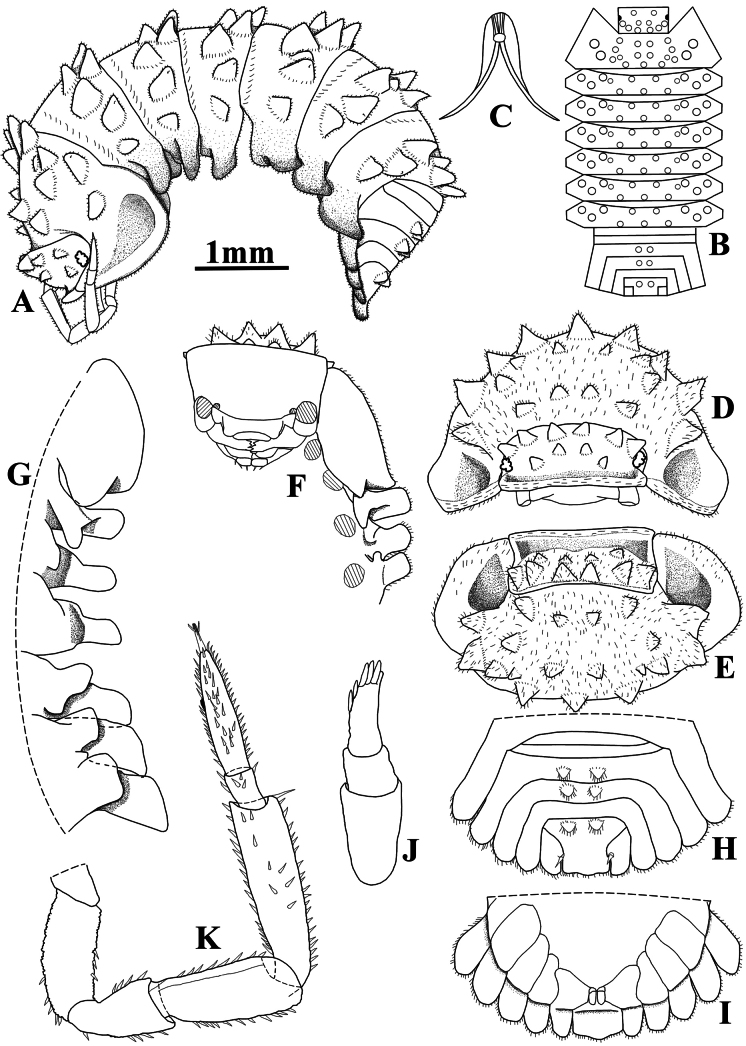
*Diploexochustroglobius* López-Orozco, Borja-Arrieta & Campos-Filho, sp. nov. (♀ paratype, CBUDC-CRU 396) **A** habitus, lateral view **B** dorsal tubercles scheme **C** dorsal scale-seta **D** cephalon and pereonite 1, dorsal view **E** cephalon and pereonite 1, posterior view **F** cephalon and pereonites 1–4, frontal view **G** pereonites 1–7 epimera, ventral view **H** pleotelson and uropods, dorsal view **I** pereonite 7, pleonites 3–5, telson, and uropods, ventral view **J** antennula **K** antenna.

**Male.** Pereopods 1–7 (Fig. 7A, B) without particular modifications. Genital papilla as in Fig. 9C. Pleopod 1 (Fig. 7C) exopod triangular, as wide as long, outer and inner margin bearing many small setae, distal and proximal outer parts triangular; endopod about twice as long as exopod. Pleopod 2 (Fig. 7D) exopod triangular, outer margin strongly concave bearing many setae; endopod slightly longer than exopod. Pleopod 3–5 exopods as in Fig. 7E–G.

##### Etymology.

The new species is named after the Vallenato music singer Diomedes Díaz Maestre, also known as “El Cacique de la Junta”.

##### Remarks.

*Diploexochuscacique* sp. nov. easily differs from the previously mentioned species in the pattern of the dorsal tubercles of the pleon and telson, the pereonites 1 and 2 epimera with ventral lobes surpassing the posterior margin of the epimera, and the club-shaped uropod endopod.

##### Natural history.

Specimens of *Diploexochuscacique* sp. nov. were collected on tree bark around the Luis Pablo Ojeda Cave (Bañaderos cave), Cerro Bañaderos, Hatonuevo, La Guajira (Fig. 8B). The area where the *D.cacique* sp. nov. species is found is composed of TDF and is part of the Sierra de Bañadero integrated management district (DMI), a mountain system in the upper basin of the Camarones River in the department of La Guajira, which is connected to the Sierra Nevada de Santa Marta.

##### Distribution.

This species is known only from the type locality at Cerro Bañaderos, Hatonuevo, La Guajira, which is included into a TDF area (Fig. 1).

#### ﻿ Diploexochus troglobius

Taxon classificationAnimaliaIsopodaArmadillidae

López-Orozco, Borja-Arrieta & Campos-Filho sp. nov.

70661144-4DB8-54F3-898B-A892503DEBD3

https://zoobank.org/0EB8C9FC-F2AD-4D8F-963F-412AE50B7586

**Figure 10.**
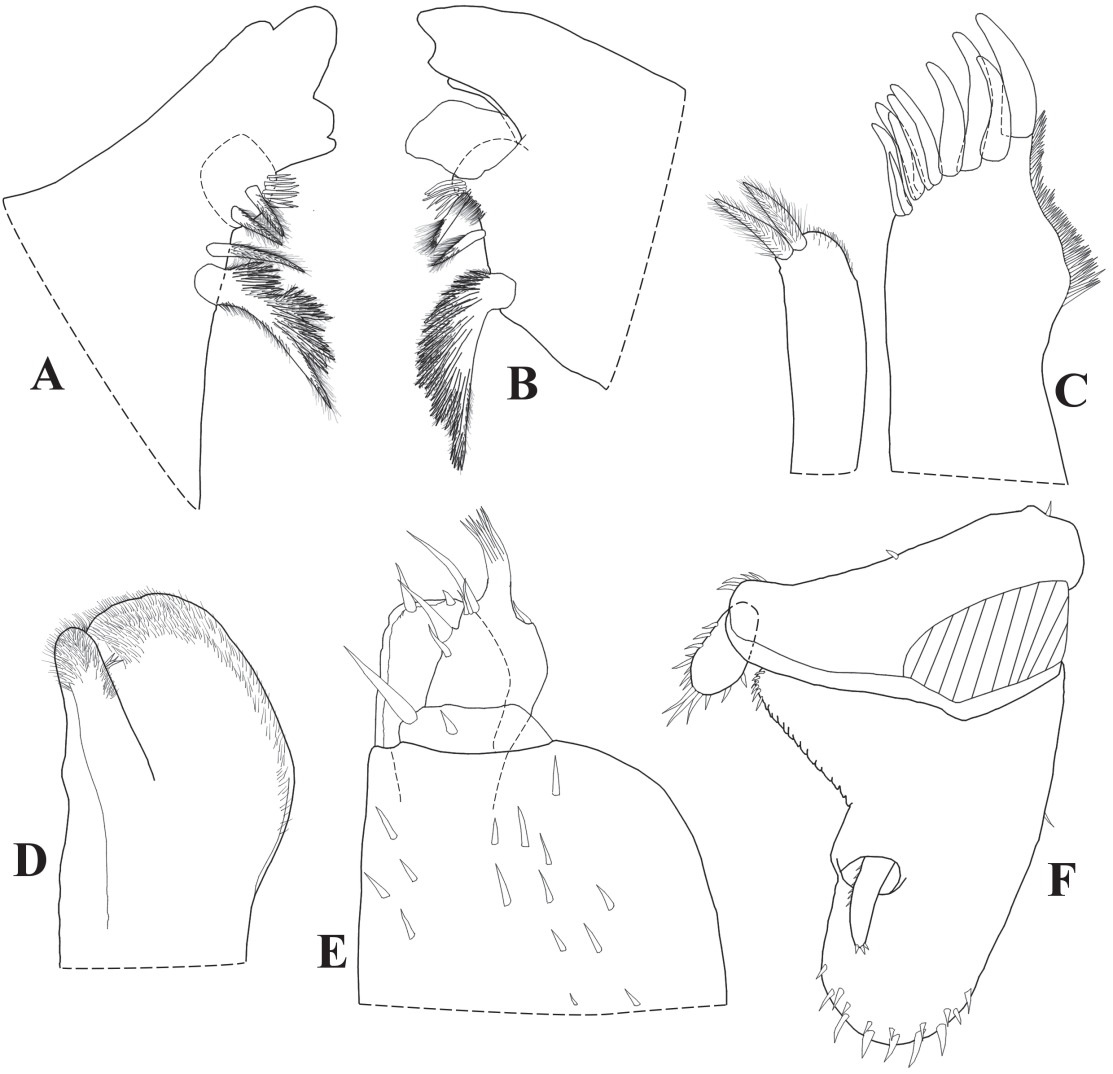
*Diploexochustroglobius* López-Orozco, Borja-Arrieta & Campos-Filho, sp. nov. (♀ paratype, CBUDC-CRU 396) **A** left mandible **B** right mandible **C** maxillula **D** maxilla **E** maxilliped **F** uropod.

**Figure 11.**
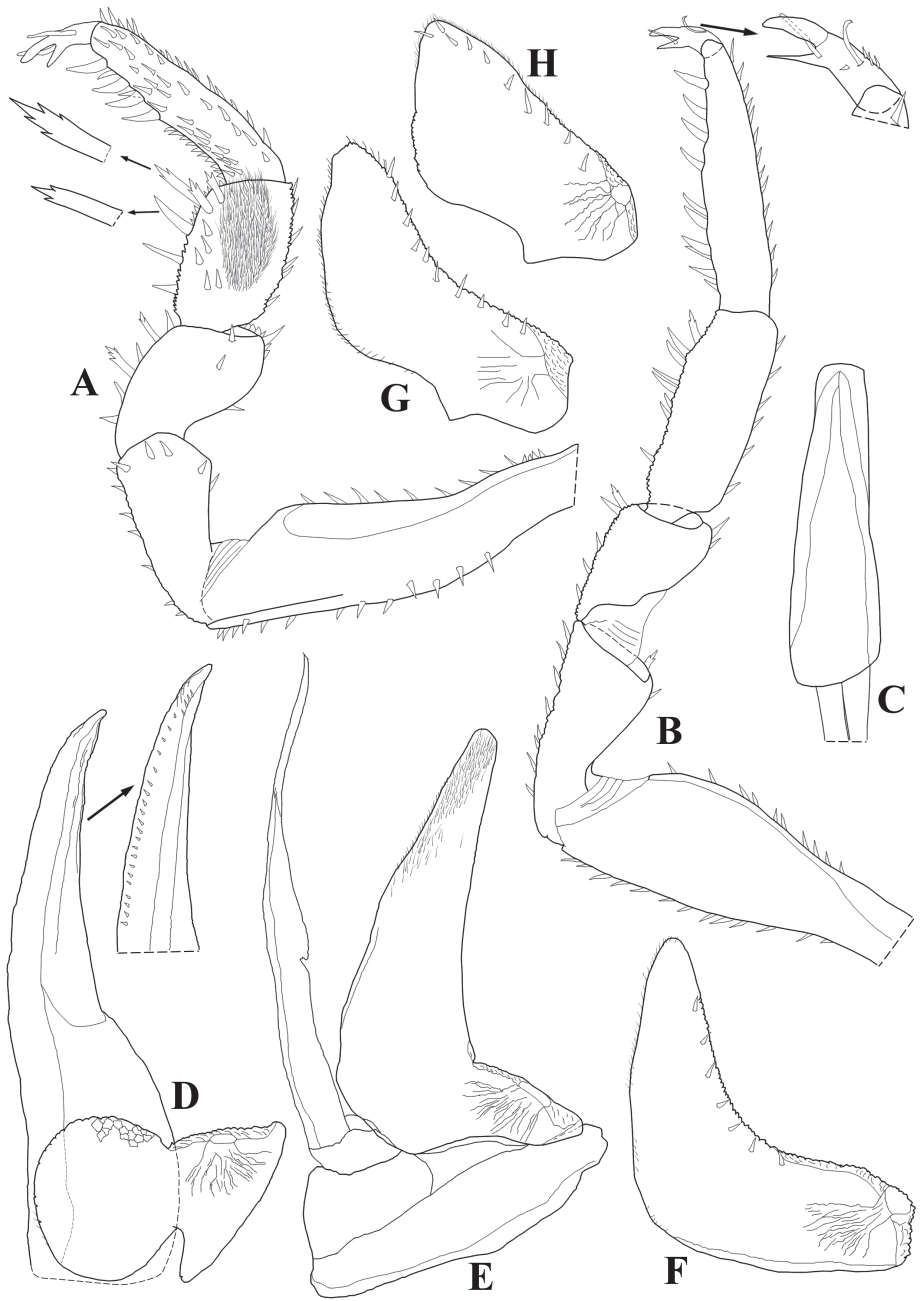
*Diploexochustroglobius* López-Orozco, Borja-Arrieta & Campos-Filho, sp. nov. (♂ paratype, CBUDC-CRU 396) **A** pereopod 1 **B** pereopod 7 **C** genital papilla **D** pleopod 1 **E** pleopod 2 **F** pleopod 3 exopod **G** pleopod 4 exopod **H** pleopod 5 exopod.

**Figure 12.**
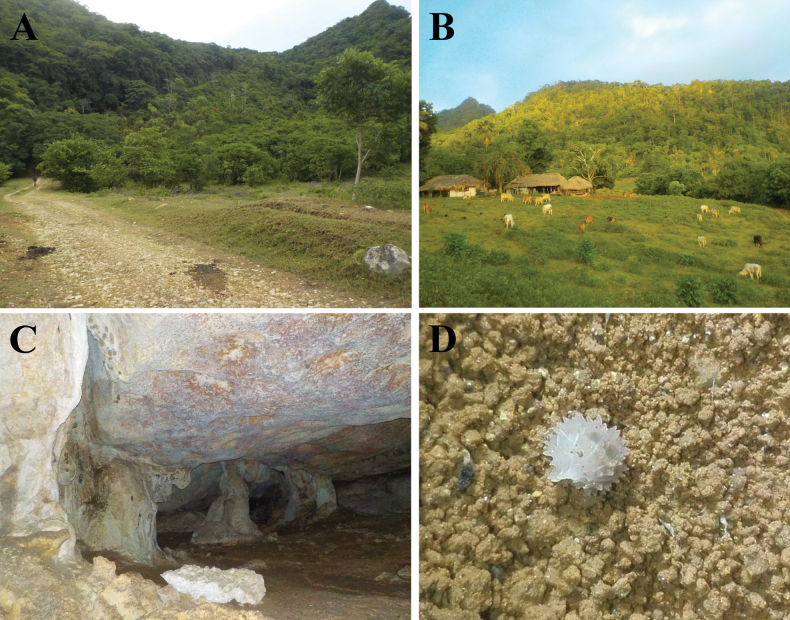
**A** Tropical Dry Forest around Roca Madre Adventure Park, Sucre **B** livestock around Roca Madre Adventure Park **C** Roca Madre Cave Gallery **D***Diploexochustroglobius* López-Orozco, Borja-Arrieta & Campos-Filho, sp. nov. under limestone rocks.

**Figure 13.**
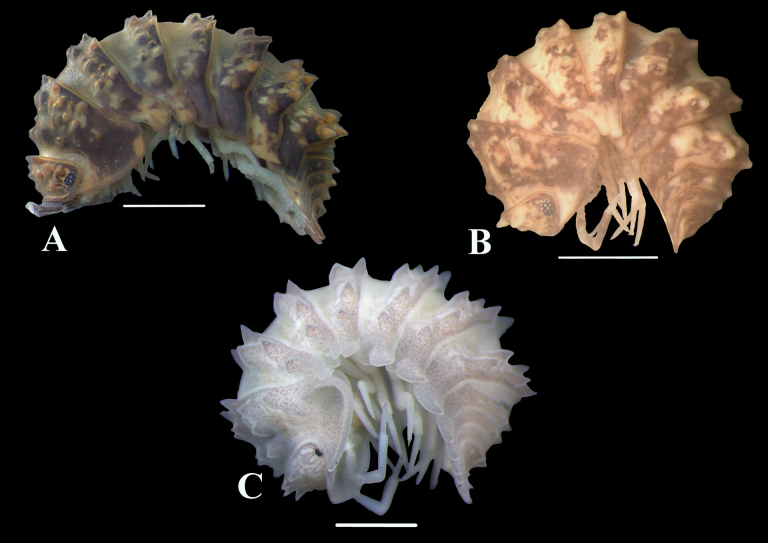
Habitus of the species of the genus *Diploexochus* Brandt, 1833 of Colombia: **A***Diploexochusbrevispinis* (Pearse, 1915), comb. nov. **B***Diploexochuscacique* López-Orozco, Carpio-Díaz & Campos-Filho, sp. nov. **C***Diploexochustroglobius* López-Orozco, Borja-Arrieta & Campos-Filho, sp. nov. Scale bars: 1 mm.

Figs 1 , 9 , 10 , 11 , 12 , 13C

##### Type material.

Colombia • 1♂, ***holotype***, Roca madre Cave, Campo Aventura Roca Madre, La Piche, Toluviejo, Sucre, 9°30'50.2"N, 75°23'36.6"W, 12.VII.2018, leg. CM López-Orozco, R Borja-Arrieta, CBUDC-CRU 393 • 1♂, ***paratypes***, same data as holotype, CBUDC-CRU 394 • 1♂, 1♀ (parts in micro-preparations), ***paratypes***, same data as holotype, CBUDC-CRU 396.

##### Description.

Maximum body length: male 2.7 mm, female 4.2 mm. Body outline as in Fig. 9A. Color faintly brown; body pigments not discernible in vivo (Fig. 12D). Endoantennal conglobation (Figs 9A, 12D, 13C). Dorsum covered with large triangular tubercles, arranged as follows (Fig. 9A, B): vertex of cephalon with 10 tubercles in two rows; pereonite 1 with 19 tubercles; pereonites 2–6 with 13 tubercles; pereonite 7 with 11 tubercles; pleonites 3, 4, and telson with two median tubercles. Pereonites 1–7 epimera with one line of ***noduli laterales*** per side inserted on outer surface of second tubercle of posterior row (Fig. 9A). Dorsal surface densely covered with elongated semi-circular scale-setae, conferring pilous aspect (Fig. 9C). Cephalon (Fig. 9D–F) with prominent frontal shield, distinctly protruding above vertex; eyes with four ommatidia. Pereonites 1–7 epimera flattened and slightly directed outwards; pereonite 1 grooved on posterior lateral margin, inner lobe of schisma rounded and extending beyond posterior margin of outer lobe. Pereonite 2 with triangular ventral lobe rounded at apex, extending beyond posterior margin of epimera; pereonites 3 and 5–7 with well-marked ventral ridge (Fig. 9A, F, G). Pleonites 3–5 epimera (Fig. 9H, I) well developed, sub-rectangular, and directed outwards, distal margins rounded. Telson (Fig. 9H) with proximal part broader than distal part, dorsum slightly depressed, distal margin sinuous. Antennula (Fig. 9J) of three articles, proximal and distal subequal in length, distal article with five stout aesthetascs inserted sub-apically. Antenna (Fig. 9K) short, not surpassing posterior margin of pereonite 1 when extended backward; flagellum of two articles, the distal about three times as long as first, bearing one row of two lateral aesthetascs. Mandibles with molar penicil semi-dichotomized; left mandible (Fig. 10A) with 2+1 penicils, right mandible (Fig. 10B) with 1+1 penicils. Maxillula (Fig. 10C) inner endite with two stout penicils, distal margin bearing thin setae; outer endite with 4+6 teeth simple. Maxilla (Fig. 10D) inner lobe rounded covered with thick setae; outer lobe rounded three times as wide as inner lobe covered with thin setae. Maxilliped basis (Fig. 10E) rectangular, bearing sparse setae; palp with two distinct setae on basal article; endite subrectangular, medial seta surpassing distal margin, distal margin bearing one seta. Pereopods 1–7 merus and carpus with sparse setae on sternal margin; carpus 1 with distal seta cleft at apex; ungual seta and dactylar organ simple not surpassing outer claw. Uropod (Fig. 10F) protopod flattened, enlarged on basal part, distal part elongated and sub-rectangular, distal margin rounded, medial margin concave with L-shaped appearance; exopod as long as endopod inserted dorsally near medial margin bellow distinct lobe, lobe not extending beyond medial margin; endopod short bearing many short setae. Pleopods 1–5 exopods with monospiracular respiratory structures.

**Male.** Pereopods 1–7 (Fig. 11A, B) without particular modifications. Genital papilla as in Fig. 11C. Pleopod 1 (Fig. 11D) exopod hour-glass shaped, twice as wide as long, inner portion rounded, outer portion triangular, distal and proximal margins narrow on middle; endopod about three times as long as exopod. Pleopod 2 (Fig. 11E) exopod triangular, outer margin strongly concave; endopod slightly longer than exopod. Pleopod 3–5 exopods as in Fig. 11F–H.

##### Etymology.

Latin: *troglo* + *bio* = cave-dwelling. The new name of the species is an adjective that refers to the troglobitic category of the species.

##### Remarks.

*Diploexochustroglobius* sp. nov. is easily distinguishable from the congeners in the arrangement of the dorsal tubercles of the pleon, dorsal surface with pilose aspect, eyes of four ommatidia, and the shape of the male pleopod 1 exopod.

##### Natural history.

Specimens of *D.troglobius* sp. nov. were collected in the aphotic zone of the Roca Madre Cave, beneath limestone rocks (Fig. 12D). This species is considered troglobitic due to the reduction of body pigments and reduction in the number of ommatidia. In addition, several surveys were conducted outside the cave and other subterranean ecosystems to confirm its restricted distribution. This species is considered endemic to the study area.

##### Distribution.

This species is known only from the type locality at Roca Madre Cave, Sucre, inserted on TDF area (Fig. 1).

### ﻿*Key to species of*Diploexochus

**Table:** 

1	Dorsal surface of the pleon with 10 tubercles	**2**
–	Dorsal surface of the pleon with < 10 tubercles	**3**
2	Pereonite 1 with 24 large, triangular, and acute tubercles	***D.echinatus* Brandt, 1833**
–	Pereonite 1 with 21 acute and rectangular tubercles	***D.obscurus* Cardoso, Bastos-Pereira & Ferreira, 2022**
3	Cephalon with 8 dorsal tubercles	**4**
–	Cephalon with 10 or more tubercles	**5**
4	Pereonites 5–7 with 12 tubercles distributed in two rows; pleonites 3 and 4 with 2 paramedian tubercles	***D.carrapicho* Campos-Filho, López-Orozco & Taiti, 2023**
–	Pereonites 5–7 with 7 tubercles distributed in a single row; pleonites 3 and 4 without paramedian tubercles	***D.exu* Campos-Filho, Sfenthourakis & Bichuette, 2023**
5	Eyes with 20 or more ommatidia	**6**
–	Eyes with 16 or fewer ommatidia	**7**
6	Antennula with nine apical aesthetascs; pereonites 2–6 with 13 tubercles distributed in 2 rows	***D.spinatus* Cardoso, Bastos-Pereira & Ferreira, 2022**
–	Antennula with 4 subapically inserted aesthetascs; pereonites 2–6 with 16 tubercles distributed in 2 rows	***D.cacique* sp. nov.**
7	Pleonites 3–4 with a single tubercle on median portion; pereonite 7 with 15 tubercles in 2 rows	***D.brevispinis* comb. nov.**
–	Pleonites 3–4 with 2 paramedian tubercles; pereonite 7 with 11 tubercles in 2 rows	***D.troglobius* sp. nov.**

## ﻿Discussion

The genus *Diploexochus* is distributed in the Neotropical region and is distinguished by the shape and direction of the epimera and pleonites, the frontal shield of the cephalon, and the arrangement of dorsal tubercles (Campos-Filho et al. 2017a, 2023a; Cardoso et al. 2023). In the illustrations of *D.obscurus* provided by Cardoso et al. (2023), pleonite 4 appears to have four tubercles. However, the description mentions that it has two paramedian tubercles, a pattern shared with the species *D.echinatus*. Therefore, a reanalysis of this species is necessary for its correct description and comparison.

The Colombian species of *Diploexochus* are distributed in TDF areas (Fig. 1), considered to host a high level of diversity and endemism, where the species are adapted to extreme environmental conditions, such as drought and extreme temperatures (Alcázar et al. 2021). However, TDF is currently one of the most threatened and least protected tropical biomes on the planet (Murphy and Lugo 1986; Janzen 1988). In Colombia, it is estimated that ~ 720,000 hectares of the original 8 million hectares of TDF remain, and only 5% are under the protection of the National System of Protected Areas (García et al. 2014; Pizano and García 2014). This reduction in vegetation cover is mainly due to various anthropogenic pressures, leading to a loss of biodiversity (Hoekstra et al. 2005; Portillo-Quintero and Sánchez-Azofeifa 2010). Therefore, studies regarding dynamics of populations, including oniscidean species, are necessary to better understand the ecological relations between species and area. Moreover, as in other parts of the world, these organisms could act model to improve conservation and sustainable programs (Solomou et al. 2019; Reboleira et al. 2022).

Subterranean systems are considered biodiversity refuges (Muñoz-Saba et al. 1998a; Culver and Sket 2000; Pipan et al. 2020). The species diversity contained in these habitats mainly depends on strict climatic conditions and energy flow (Ardila 2006; Castellanos-Morales 2018). However, this emerging biodiversity is primarily threatened by the alteration of surrounding natural habitats, anthropogenic pressure, uncontrolled tourism, and mining, among other factors (Restrepo 2007, 2011; Manco et al. 2017; Angarita 2018; Zafra 2021). In Colombia, more than 500 subterranean systems located in 12 biospeleological provinces are known (Muñoz-Saba et al. 1998a, 1998b; Valdivieso 2022). Despite this number, biodiversity research is scarce and isolated for some areas or departments of the country (Muñoz-Saba et al. 1998b, 2013; Vides-Navarro et al. 2015; Angarita 2018; Barriga et al. 2019). Furthermore, most of the country’s caves are distributed along transformed agro-ecosystems without any protection (Muñoz-Saba et al. 1998a, 2013). Recently, the National Government enacted the Law number 2237 of 2022, assuring the protection of the Colombian speleological heritage, where environmental authorities will declare protected areas that include speleological biodiversity. However, efforts to conserve these fragile ecosystems are not sufficient, since the number of subterranean ecosystems is still under estimation and the biotic composition, which are sensitive to anthropogenic disturbances, is mostly unknown. It is worth mentioning that this component probably hosts a high degree of endemic species and specific microhabitats or substrates as observed in other studies (Campos-Filho et al. 2017a, 2017b, 2018; López-Orozco et al. 2024a, 2024b). Thus, more studies are needed to assess the conservation status of these ecosystem and quantify and identify its diversity for better conservation efforts.

Regarding the knowledge of oniscideans in Colombian caves, only the species *Ctenorillopapagayoensis* Carpio-Díaz, Borja Arrieta & Campos-Filho, 2023, has been described from the Cueva de Los Papagayos in the department of Santander; *Ctenorillobinomio* Carpio-Díaz, Bichuette & Campos-Filho, 2023, for the Cueva de San Miguel in the department of Bolívar; *Pulmoniscusturbanaensis* López-Orozco, Carpio-Díaz & Campos-Filho, 2017; and *Porcellionidespruinosus* (Brandt, 1833) for the Cueva La Mojana in the department of Atlántico (Carpio-Díaz et al. 2023a, 2023b). Additionally, the genera *Colomboniscus* Vandel, 1972, *Colomboscia* Vandel, 1972, *Sphaeroniscus* Gerstäcker, 1854, *Neosanfilippia* Brian, 1957 (Scleropactidae Verhoeff, 1938), and *Ischioscia* Verhoeff, 1928 (Philosciidae Kinahan, 1857) have been reported from the Cueva de los Papagayos in Santander. The data from the study also include Cueva de Los Indios and Hoyo del Aire in the department of Santander (Sket 1988; Castellanos-Morales et al. 2015; Barriga et al. 2019), although these records do not include species formal descriptions. Regarding other taxa, five studies related to arachnids and a biological inventory of some orders in the Caribbean region have been published (Armas et al. 2015; Cala-Riquelme et al. 2015; Torres-Contreras et al. 2015; Vides-Navarro et al. 2015; García et al. 2022; Moreno-González et al. 2023). This work represents the first record of a troglobitic terrestrial isopod for both the genus and the country, increasing the number of species in the national inventory.

The species *Diploexochustroglobius* sp. nov. is recorded only from Roca Madre cave and has a low population density, supporting both the cave and the species suitable for conservation. Similarly, species such as *Charinusrocamadre* Torres-Contreras, Álvarez García & De Armas, 2015 and *Heterophrynuscaribensis* De Armas, Torres-Contreras & Álvarez García, 2015 (Amblypygi, Charinidae) are under ecological stress due to cattle (Fig. 12B), deforestation, mining, and uncontrolled ecotourism (vandalism). This demonstrates the need to formulate and implement conservation strategies by governmental entities including the scientific community and the general public in decision-making processes.

In the last years, the study of the oniscofauna from Colombia has increased, of which more than 30 epigean species have been described, consolidating a total of 73 species distributed throughout the territory (e.g. López-Orozco et al. 2014, 2016, 2017, 2022; Carpio-Díaz et al. 2016, 2018, 2021, 2023a, 2023b; Campos-Filho et al. 2020; Bravo-Rodríguez et al. 2024). However, the biodiversity of the cave-dwelling isopod species is a new topic and considering the number of caves, this diversity is far from complete.

## Supplementary Material

XML Treatment for Diploexochus

XML Treatment for Diploexochus brevispinis

XML Treatment for Diploexochus cacique

XML Treatment for Diploexochus troglobius

## ﻿Additional information

### Author ORCIDs

Ricardo Borja-Arrieta https://orcid.org/0000-0002-5064-5080

Carlos Mario López-Orozco https://orcid.org/0000-0002-3251-7739

Yesenia M. Carpio-Díaz https://orcid.org/0000-0001-5116-9736

Miguel Gutierrez-Estrada https://orcid.org/0000-0003-2017-4262

Ivanklin Soares Campos-Filho https://orcid.org/0000-0001-6139-8241

Gabriel R. Navas-S. https://orcid.org/0000-0001-9554-6345

Maria Elina Bichuette https://orcid.org/0000-0002-9515-4832

### Data availability

All of the data that support the findings of this study are available in the main text.
